# Advances in the research of exosomes in renal cell carcinoma: from mechanisms to applications

**DOI:** 10.3389/fimmu.2023.1271669

**Published:** 2023-10-24

**Authors:** Rui Rui, Liqun Zhou, Shiming He

**Affiliations:** ^1^ Department of Urology, Peking University First Hospital, Beijing, China; ^2^ The Institution of Urology, Peking University, Beijing, China; ^3^ Beijing Key Laboratory of Urogenital Diseases (Male) Molecular Diagnosis and Treatment Center, Beijing, China; ^4^ National Urological Cancer Center, Beijing, China

**Keywords:** tumor-derived exosomes, extracellular vesicles, renal cell carcinoma, tumor biomarkers, lipid biopsy

## Abstract

Renal cell carcinoma (RCC) is one of the most malignant urological tumors. Currently, there is a lack of molecular markers for early diagnosis of RCC. The 5-year survival rate for early-stage RCC is generally favorable; however, the prognosis takes a significant downturn when the tumor progresses to distant metastasis. Therefore, the identification of molecular markers for RCC is crucial in enhancing early diagnosis rates. Exosomes are a type of extracellular vesicle (EV) typically ranging in size from 30 nm to 150 nm, which contain RNA, DNA, proteins, lipids, etc. They can impact neighboring receptor cells through the autocrine or paracrine pathway, influence cellular communication, and regulate the local immune cells, consequently shaping the tumor immune microenvironment and closely associating with tumor development. The clinical application of exosomes as tumor markers and therapeutic targets has ignited significant interest within the research community. This review aims to provide a comprehensive summary of the advancements in exosome research within the context of RCC.

## Introduction

Exosomes, a crucial subset of extracellular vesicles (EVs), typically range between 30 nm and 150 nm in diameter and transport a variety of biomolecules such as lipids, non-coding RNA, and proteins. Exosomes were first observed during maturation of sheep reticular cells (1). Exosomes primarily originate from cellular endocytosis. They are formed through the invagination of the limiting membrane of endosomes, leading to the creation of intraluminal vesicles (ILVs) within endosomes. These ILVs are eventually released from the cell as exosomes when multivesicular bodies fuse with the plasma membrane (2). The role of exosomes was originally thought to be the secretion of unneeded components into the extracellular space. As our understanding of exosomes grows, their high heterogeneity becomes evident because of their diverse cellular origins. Zhang et al. (3) identified two subpopulations of exosomes: large exosomal vesicles, 90 nm to 120 nm, Exo-L; and small exosomal vesicles, 60 nm to 80 nm, Exo-S. Exosomes facilitate intercellular communication and impact the homeostasis of the internal environment, playing pivotal roles in various diseases and tumors. Notably, exosomes exhibit significant heterogeneity, even among the same cell types, depending on environmental factors like oxygen tension, cell morphology (4), or cellular stimuli from apoptotic or autophagic signaling (5). Furthermore, the protein composition of specific EV subgroups varies in response to different agonists (6).

As exosome research advances, its role in tumor development has garnered significant attention. Exosome-derived RNA, mRNA, DNA, proteins, and metabolites can alter the fate of receptor cells via autocrine or paracrine pathways. For example, exosomes derived from chronic myeloid leukemia (CML) cells carry transforming growth factor-β (TGFβ1), which, in an autocrine manner, binds to the TGFβ1 receptor on CML cells, promoting tumor growth through activation of extracellular regulated MAP kinase (ERK), Akt kinase (AKT), and anti-apoptotic pathways (7). In breast cancer, miR-9 derived from breast cancer cells exerts paracrine effects on mammary fibroblasts, affecting their intrinsic properties and regulating transition of human mammary fibroblasts into cancer-associated fibroblasts (CAFs), ultimately fostering tumor growth (8).

Renal cell carcinoma (RCC) arises from the epithelial cells of renal tubules and stands as the third most common tumor within the urinary system, constituting approximately 90% of all malignant kidney tumors (9). Kidney cancer has an insidious onset, and reducing cancer mortality remains a major goal of the scientific community. Currently, the morbidity and mortality of clear cell RCC (ccRCC) continue to increase worldwide. Despite notable advancements in recent years in our understanding of ccRCC, the prognosis for this condition continues to be disheartening. Whereas the majority of tumors are considered benign, approximately one-third of them can become malignant, carrying a risk of metastasis as high as 70% (10). More than 60% of ccRCC cases are incidentally discovered. Despite improvements in imaging techniques, more than 30% of patients still present with tumor metastases, particularly in the bones and lungs, accounting for approximately 15% of all cases at the time of diagnosis (11). Therefore, the clinical challenge lies in identifying biomarkers and developing prognostic models for metastatic and advanced RCC to enhance treatment options and realize the goal of personalized oncology (12). Diagnostic biomarkers aid in disease detection, whereas prognostic biomarkers offer insights into disease progression and recurrence. Although abdominal ultrasound is a commonly used clinical screening method for renal cancer, its effectiveness in early diagnosis, particularly for small tumors, is limited. Valuable molecular markers for early screening or diagnosis of renal cancer have not yet been reported. The identification of reliable molecular markers could significantly reduce the number of cases detected at an advanced stage with a poor prognosis. Moreover, in cases where early detection is not feasible, biomarkers predicting treatment response can facilitate the timely initiation of second-line therapies. In addition, the scarcity of validated prognostic biomarkers poses a challenge in guiding the selection of optimal treatment approaches. Biomarkers encompass various categories, including nucleic acids, proteins, metabolites, isoenzymes, and hormones, and are typically classified as diagnostic, prognostic, or predictive (13).

## Exosomes and the development of RCC

Exosomes play a pathologic role in renal carcinogenesis and metastasis. Exosome-derived non-coding RNAs in RCC can modulate the epithelial–mesenchymal transition (EMT) pathway in RCC cells, thereby promoting the development and metastasis of RCC (14). In addition, exosomes modulate the tumor immune microenvironment, facilitating immune escape (15). Furthermore, they also play a role in regulating various physiological processes, including tumor growth, angiogenesis, metastasis, drug resistance, and stem cell maintenance (16, 17). Wang et al (14). reported that cancer stem cell–derived miR-19b-3p promotes ccRCC metastasis by regulating the expression of EMT-related genes E-Cadherin, N-Cadherin, Vimentin, and twist in ccRCC cells. Hypoxia could induce the expression of induce the expression of hypoxia-induced long non-coding RNA associated with RCC (lncHILAR), which functions as a competing endogenous RNA (ceRNA) for miR-613/206/1-1-1-3p and competitively activates the Jagged1/Notch/CXCR4 pathway, thereby promoting invasion and migration of RCC *in vitro* and metastasis *in vivo*. In addition, hypoxic RCC can simultaneously secrete exosomes containing lncHILAR, which are internalized by normoxic RCC, enhancing their invasion phenotype through the acquisition of exogenous lncHILAR (18). Tsuruda et al. (19) reported the oncogenic effects of RAB27B, an exosome-secreted protein, in renal cancer cell lines, although the specific role of RAB27B in exocytosis has not yet been determined. Circular protein kinase C iota (Circ-PRKCI) is a microRNA (miRNA) sponge, and it has been shown that Circ-PRKCI can participate in tumor progression by stimulating migration and proliferation of esophageal squamous cell carcinoma cells through sponging miR-3680-3p (20). Qian et al. (21) demonstrated that the RCC cell line Caki-1–derived exosome circ-PRKCI promotes RCC proliferation by regulating the miR-545-3p/CCND1 axis. The expression level of miR-30c-5p in urinary exosomes of patients with ccRCC was lower than that of healthy controls. Overexpression of miR-30c-5p could inhibit the growth of renal clear cell carcinoma cells, suggesting that miR-30c-5p plays a role in the development of RCC (22). ApoC1 expression was upregulated in RCC samples and increased with ccRCC progression. ccRCC cells secrete ApoC1-containing exosomes that metastasize to vascular endothelial cells and promote ccRCC cell metastasis through activation of STAT3 (23).

## Exosomes and drug resistance of RCC

Tyrosine kinase inhibitors (TKIs) were initially shown to be effective treatments for RCC. However, after a median of 14 months of treatment, the vast majority of patients develop drug resistance. Resistance to TKI therapy in RCC is also one of the major causes of post-treatment relapse (24). Exosomes are also important carriers for the propagation of cancer drug resistance (CDR), transferring drug-resistant mediators from donor cells to recipient cells, resulting in the acquisition of the CDR phenotype by previously drug-sensitive cells. Several mediators associated with drug resistance in cancer cells have been found to be transferred via exosomes, including drug efflux pumps (e.g., P-glycoprotein, ATP-binding cassette transporter G2, and ABCG2), miRNAs, lncRNAs, and other regulators such as transient receptor potential cation channel subfamily C member 5 (25).

Qu et al. (26) identified an uncharacterized lncRNA, activated in RCC with sunitinib resistance (lncARSR), that is highly expressed in sunitinib-resistant RCC and is functionally required for the RCC resistance phenotype. lncARSR could be secreted from drug-resistant cells via exosomes that convert sunitinib-sensitive cells into drug-resistant cells, thereby propagating drug resistance. Moreover, lncARSR promotes sunitinib resistance by competitively binding to miR-34 and miR-449, resulting in upregulation of AXL receptor tyrosine kinase-receptor tyrosine kinase mesenchymal-epithelial transition factor (AXL/c-MET) expression and activation of signal transducer and activator of transcription 3 (STAT3), AKT, and ERK signaling pathways. In contrast, activated AKT promotes lncARSR expression by inhibiting the transcription factors forkhead box, subgroup O 1 (FOXO1) and FOXO3a, creating a positive feedback loop in sunitinib-resistant renal cancer cells (26). Tipifarnib blocks exosome production and secretion in sunitinib-sensitive and sunitinib-resistant cells and downregulates PD-L1 expression by inhibiting endosomal sorting complex required for transport (ESCRT)-dependent and non–ESCRT-dependent exosome–endosome sorting complexes (27). Cancer cells frequently decrease DNA repair pathways and increase endogenous sources of mutation, which drive mutations, and therefore tend to be more dependent on a subset of DNA repair pathways to survive DNA damage. Targeting key DNA repair members such as poly(ADP-ribose) polymerases (PARPs) is a therapeutic strategy for cancer (28). Inhibition of PARPs by small-molecule compounds disrupts the ability of cancer cells to survive sustained DNA damage and leads to cell cycle arrest and/or cell death; however, PARP inhibitors (PARPis) have failed to significantly benefit patients, suggesting the need to develop new strategies. Liu et al. (29) reported that exosome component 1 (EXOSC1) sensitizes RCC cells to PARPis, suggesting that PARP inhibition may be an effective strategy for the treatment of Kidney renal clear cell carcinoma (KIRC) patients with high EXOSC1. EXOSC1 is associated with poorer poorer progression-free survival (PFS) and overall survival (OS) in patients with RCC, supporting its potential pathological significance.

## Exosomes and the TME of RCC

**Figure 1:**
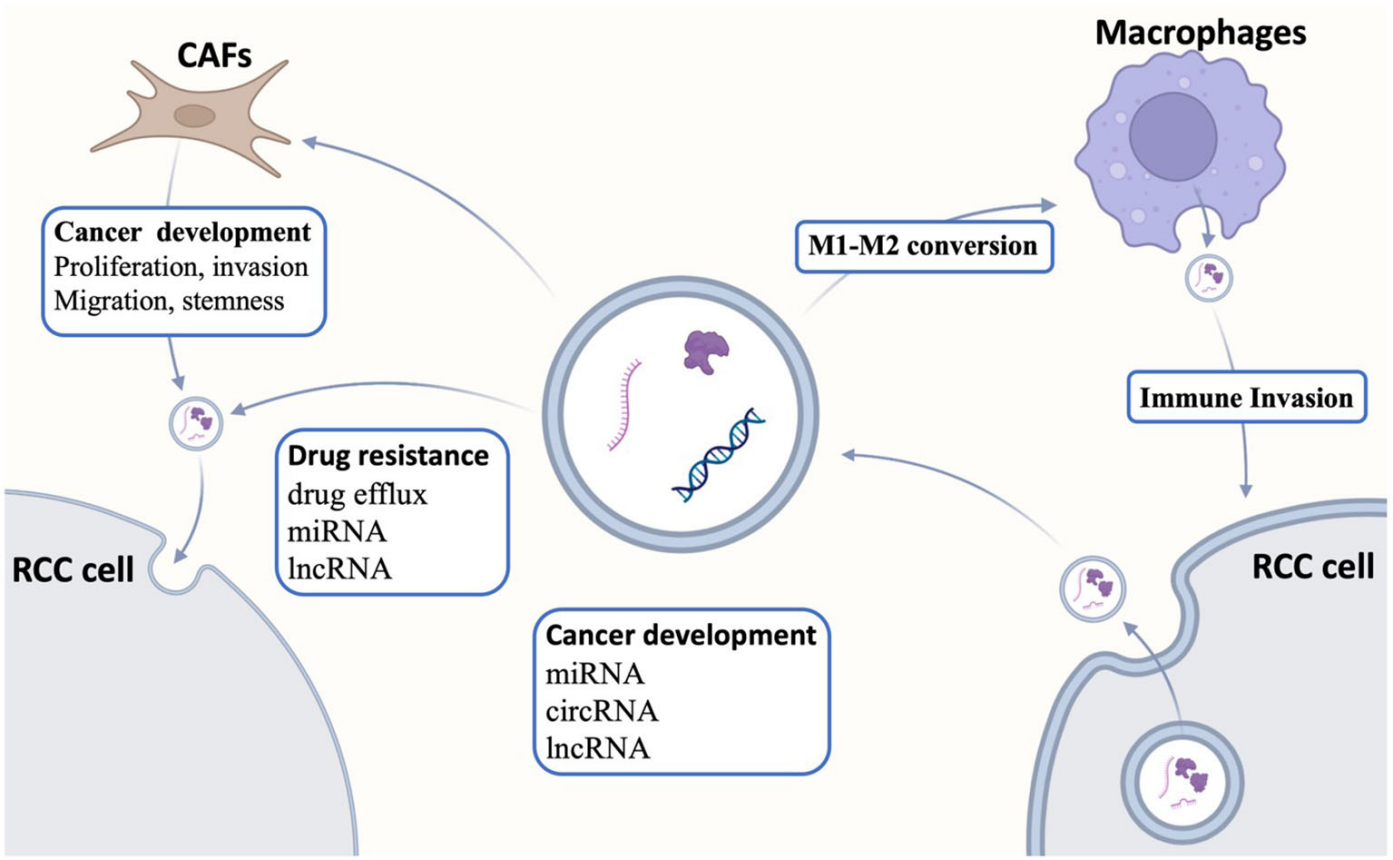
Interactions of exosomes with the tumor microenvironment. Exosomes regulate tumor differentiation, proliferation, apoptosis, and drug resistance by transmitting intercellular information, whereas exosomes are also related to the tumor microenvironment. Exosomes secreted by tumor cells can act on tumor-associated macrophages to regulate macrophage polarization, whereas exosomes secreted by tumor-associated fibroblasts regulate tumor development. CAFs, cancer-associated fibroblasts; RCC, renal cell carcinoma.

In addition to simply affecting tumor cells, exosomes also affect the immune microenvironment of the tumor (**Figure 1**). Tumor-infiltrating macrophages exhibit two distinct polarization states, termed M1 and M2. RCC-derived exosomes carry a large amount of lncARSR, which interacts with local macrophages and activates the STAT3 pathway in macrophages, leading to the conversion of macrophages from M0/1 to M2, and this polarized state results in the secretion of large quantities of anti-inflammatory factors [e.g., interleukin-10 (IL-10) and TGFβ1] by macrophages to create a local microenvironment more suited for tumor metastasis and the formation of pre-metastatic lesions (30). In addition to tumor cell–derived exosomes regulating immune cells in the tumor microenvironment, immune cells also secrete exosomes to act on tumor cells. Liu et al. (31) reported that tumor-associated macrophage-derived exosome-loaded miR-193a-5p promotes ccRCC vasculogenic mimicry and immune invasion by inhibiting TIMP metallopeptidase inhibitor 2 (TIMP2) transcription through specific sponging the 3' untranslated regions (3′UTR) of TIMP2. Moreover, the RCC-derived exosome circSAFB2 mediates M2-macrophage polarization through the miR-620/JAK1/STAT3 axis, thereby promoting ccRCC immune escape and metastasis (32). lncRNA AP000439.2 was highly enriched in ccRCC-exo. ccRCC-exo significantly activated macrophage conversion to M2-type phenotype, which led to increased expression levels of TGFβ and IL-10 in macrophages, which, in turn, promoted the migration of ccRCC cells, suggesting that exosome AP000439.2 may be a new therapeutic target for ccRCC (33). In TME, in addition to tumor cell–derived exosomes that can act on and regulate immune cells, other cellular components in TME can also secrete exosomes to modulate tumor cells. CAF-derived exosomes can smoothly enter and be internalized by cancer cells. After co-culture of cancer cells with CAF-derived exosomes, cell proliferation, migration, and invasion were significantly enhanced, and apoptosis was reduced. In addition, the expression of fibronectin, matrix metalloproteinase 9 (MMP9), MMP2, and N-calmodulin and waveform protein in the EMT pathway was elevated in cancer cells, whereas the expression of E-calmodulin was decreased and the proportion of S-phase cells was increased, which proved that CAF-derived exosomes could promote the progression of RCC (34). Moreover, miR-224-5p in the exosomes secreted by CAFs can enter ccRCC cells and regulate the proliferation, migration, invasion, and apoptosis of ccRCC cells, which, in turn, promotes the malignant behaviors of ccRCC cells, suggesting that miR-224-5p has the potential to serve as a therapeutic target for ccRCC (35). Furthermore, enrichment of CAFs was positively correlated with tumor stemness and RCC progression, and exosomal miR-181d-5p delivered by CAFs directly targeted ring finger protein 43 (RNF43) and activated the Wnt/β-catenin signaling pathway, thereby enhancing tumor stemness and mediating RCC progression (36).

## Exosomes as biomarkers of RCC

**Figure 2:**
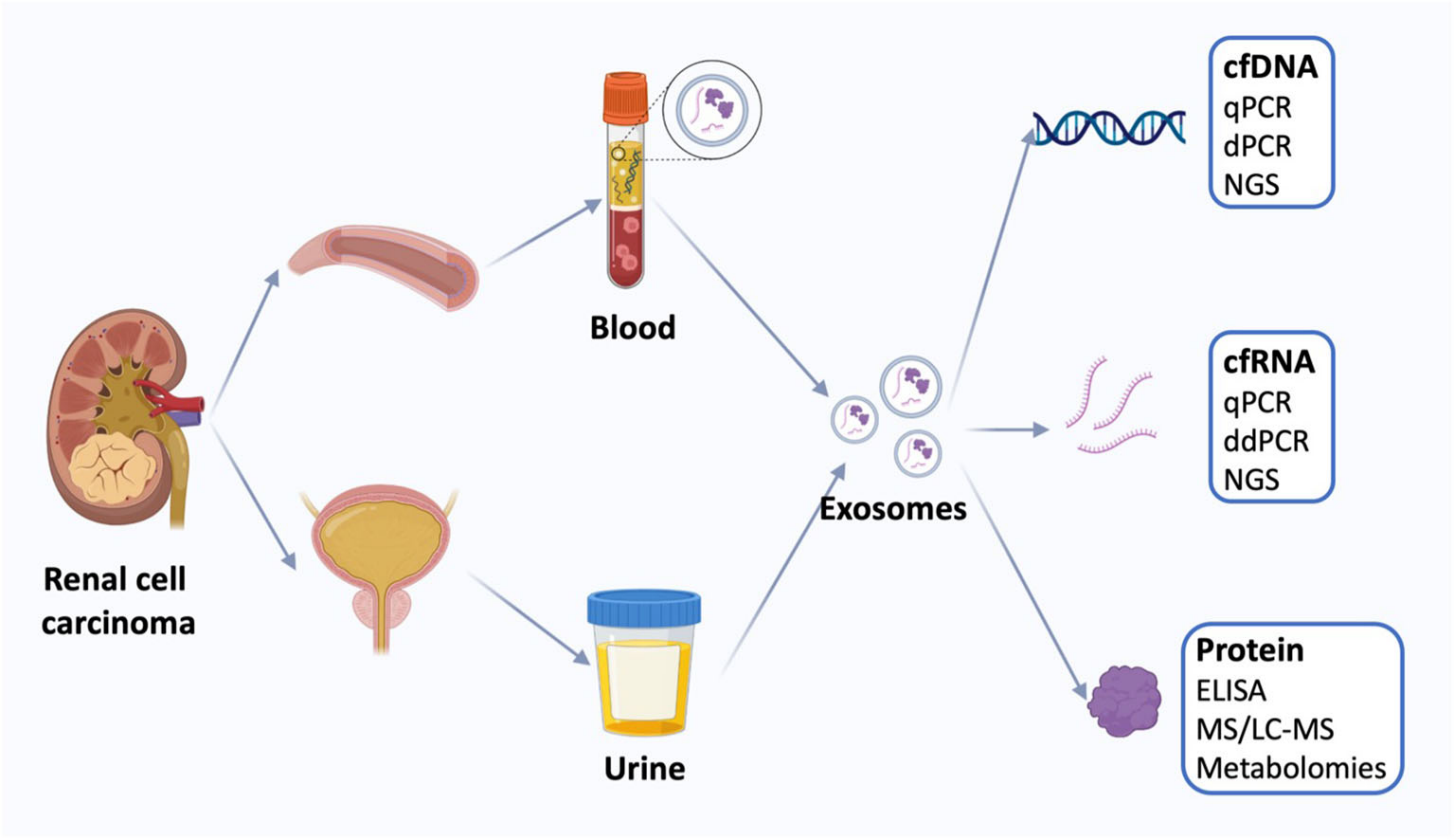
Liquid biopsy of renal cell carcinoma. Liquid biopsies for kidney cancer use mainly blood and urine samples, including exosomes and the cell-free DNA (cfDNA), cell-free RNA (cfRNA), and proteins. Quantification of circulating tumor markers at a single time point may be useful for disease staging and prognosis using a variety of techniques. cfDNA, cell-free DNA; qPCR, quantitative polymerase chain reaction; dPCR, digital polymerase chain reaction, NGS, next-generation sequencing; cfRNA, cell-free RNA; ddPCR, droplet digital polymerase chain reaction; ELISA, enzyme-linked immunosorbent assay; MS, Mass spectrometry; LS-MS, liquid chromatography-MS.

RCC has the highest mortality rate among genitourinary malignancies. Unfortunately, around 30% of patients are already in the metastatic stage at the time of diagnosis. Although localized RCC can often be cured with surgery, the prognosis dramatically worsens once metastasis occurs. Currently, aside from invasive biopsy, which often yields limited results, there is a notable absence of diagnostic tools for confirming renal masses. As a result, there is an urgent demand for non-invasive molecular testing of urine or serum as a viable alternative to biopsy (37). Although tissue biopsy remains the gold standard for tumor diagnosis, it is limited in that it reflects a single point in time at a specific tumor site. The emergence of next-generation sequencing technology has paved the way for the discovery of novel, more comprehensive, and less invasive biomarkers, bringing us closer to the realization of precision cancer medicine. Minimally invasive testing, commonly known as “liquid biopsy” (**Figure 2**), typically involves the analysis of circulating tumor cells, circulating tumor DNA, and tumor-derived exosomes to access tumor genomic signatures (38).

Recent studies have shown that EV RNA may be potential biomarkers for prostate cancer (39), hepatocellular carcinoma (40), and pancreatic ductal adenocarcinoma (41). In a single-center retrospective study, researchers collected serum from 106 patients with renal clear cell carcinoma and 73 patients with benign renal tumors and constructed a diagnostic tag for renal clear cell carcinoma by screening three candidate genes through multivariate stepwise logistic regression analysis. The diagnostic tag can differentiate between renal masses of indeterminate nature, such as cystic renal carcinomas, angiomyolipomas of lack of fat, and renal eosinophilic cell tumors, especially when the mass is small and imaging does not allow for identification of the tumor substance (42). In a case study containing 16 patients with ccRCC and healthy subjects, miR-30c-5p in urinary exosomes was the only one that was not differentially expressed in other urologic tumors, which demonstrated the specificity of its expression in ccRCC. Urinary exosomes of patients with ccRCC showed lower expression levels of miR-30c-5p than normal subjects, and overexpression of miR-30c-5p could inhibit the growth of ccRCC cells and tumorigenicity in nude mice, suggesting that miR-30c-5p is associated with the development of renal clear cell carcinoma (43). Bao et al. (44) identified six key miRNAs and developed a programmed necrosis–related miRNA signature to predict the prognosis of ccRCC. Exosome-derived nucleic acids can be used as therapeutic markers for RCC prognosis in addition to biomarkers for RCC diagnosis. Wang et al. (45) demonstrated that miR-210 is a good prognostic biomarker, and patients with renal cancer overexpressing miR-210 have higher chances of disease recurrence and shorter survival time. In a prospective study of 35 patients treated with immunotherapy, blood was drawn before and after treatment with immune checkpoint inhibitors. miRNA-146a levels were increased after treatment, and miRNA-126 levels were decreased compared with those before treatment, suggesting that miRNA-146a and miRNA-126 may serve as a predictor of the efficacy of ICI treatment (46).

At present, many mechanism of exosomes in RCC have been reported, and a variety of ways to isolate exosomes have been developed, suggesting that liquid biopsy holds promising prospects for the diagnosis of RCC. The mechanisms of exosome-promoted RCC progression and the ways of exosome isolation have been summarized in **Table 1**.

**Table 1:** The role of exosomes in the development and diagnosis of renal cell carcinoma.

EVs origin	Separation method	Sample	Cargo type	Specific biomolecule	Potential effect	Reference
Cancer stem cells of ccRCC	Differential centrifugation	Cell culture medium	miRNAs	miR-19b-3p	1. Promote proliferation of ccRCC cells and accelerate the progress of EMT 2. CD103+ exosomes functions as a potential metastatic diagnostic biomarker	(14)
RCC cells	Polymer-based precipitation (commercialized reagent kits)	Cell culture medium	lncRNAs	lncHILAR	1. Knockdown of lncHILAR inhibited cell invasion and migration, and vice versa. 2. lncHILAR functions via the miR-613/206/1-1-3p/Jagged-1/Notch/CXCR4 axis	(18)
RCC cells	Differential centrifugation	Cell culture medium	N/A	N/A	1. No specific effect of RAB27B on exosomal function was revealed in RCC cells in regard to both quantity and function.	(19)
Patients with ccRCCs/RCC cells	Polymer-based precipitation (commercialized reagent kits) /Differential centrifugation	Serum/Cell culture medium	circRNAs	circ-PRKCI	1. circ-PRKCI promoted the proliferation of RCC via the miR-545-3p/CCND1 signaling pathway	(21)
Patients with ccRCCs	Differential centrifugation	Urine	miRNAs	miR-30c-5p	1. urinary exosomal miR-30c-5p acts as a potential diagnostic biomarker of early-stage ccRCC and may be able to modulate the expression of HSPA5	(22)
RCC cells	Differential centrifugation	Cell culture medium	lncRNAs	lncARSR	1. Intercellular transfer of lncARSR by exosomes disseminates sunitinib resistance 2. lncARSR acts as a ceRNA for miR-34 and miR-449 to promote AXL and c-MET expression	(26)
RCC cells	Differential centrifugation	Cell culture medium	N/A	N/A	1. Exosomes carry bioactive oncogenic cargos that reprogram target cells to promote tumor growth, migration, metastasis, immune evasion, and chemotherapy resistance	(27)
RCC cells	Polymer-based precipitation (commercialized reagent kits)	Cell culture medium	lncRNAs	lncARSR	1. RCC-derived exosomes facilitate the development of tumor through inducing macrophage polarization via transferring lncARSR	(30)
Tumor-associated macrophages	Differential centrifugation	Cell culture medium	miRNAs	miR-193a-5p	1. miR-193a-5p from TAM-derived exosomes downregulates the TIMP2 gene to facilitate the development of RCC	(31)
RCC cells	Differential centrifugation	Cell culture medium	circRNAs	circSAFB2	1. circSAFB2 leads to immune escape from RCC by mediating M2 macrophage polarization via the miR-620/JAK1/STAT3 axis	(32)
RCC cells	Differential centrifugation	Cell culture medium	lncRNAs	AP000439.2	1. Exosomes from ccRCC deliver AP000439.2 to promote M2 macrophage polarization via STAT3, thus enhancing ccRCC progression	(33)
Cancer-associated fibroblasts	Polymer-based precipitation (commercialized reagent kits)	Cell culture medium	N/A	N/A	1. CAF-derived exosomes are internalized into ccRCC cells and promote the progression of ccRCC.	(34)
Cancer-associated fibroblasts	Polymer-based precipitation (commercialized reagent kits)	Cell culture medium	miRNAs	miR-224-5p	1. miR-224-5p can enter ccRCC cells via CAF-derived exosomes, promoting the malignant behaviors of ccRCC cells.	(35)
Cancer-associated fibroblasts	Polymer-based precipitation (commercialized reagent kits)	Cell culture medium	miRNAs	miR-181d-5p	1. Exosomal miR-181d-5p transferred from CAFs to RCC cells directly suppressed the expression of RNF43 and activated Wnt/β-catenin signaling pathway	(36)
Patients with ccRCCs	Polymer-based precipitation (commercialized reagent kits)	Serum	mRNAs	CUL9, KMT2D, PBRM1, PREX2, and SETD2,	1. A differential diagnostic signature that comprised CUL9, KMT2D, and PREX2 for RCC detection was developed	(42)
Patients with ccRCCs	Polymer-based precipitation (commercialized reagent kits)	Serum	miRNAs	miR-210 and miR-1233	1. Circulating levels of exosomal microRNAs miR-210 and miR-1233 have potential as biomarkers for diagnostic and monitoring purposes in renal cancer	(43)
Patients with ccRCCs/RCC cells	Polymer-based precipitation (commercialized reagent kits)	Serum/Cell culture medium	miRNAs	miR-210	1. Serum exosomal miR-210 originating from tumor tissue has potential as a novel non-invasive biomarker for the detection and prognosis of ccRCC.	(45)
Patients with ccRCCs	N/A	Plasma	miRNAs	miR-146a and miR-126	1. miR-146a and miR-126 can be used as predictors for immune checkpoint inhibitor therapy effectiveness	(46)

N/A, not applicable; ccRCC, clear cell renal cell carcinoma; RCC, renal cell carcinoma; CAFs, cancer-associated fibroblasts; ceRNA, competing endogenous RNA; lncRNA, long non-coding RNA; miRNA, microRNA; circRNA, circular RNA.

## Discussion

In recent years, an increasing number of research studies have elucidated the physiological functions of exosomes that are critical to understand how they influence cancer development. Exosomes could activate signaling pathways through intercellular communication to promote cancer cell proliferation. In addition, exosomes are involved in many processes of tumor metastasis, including epithelial–mesenchymal transition, promotion of cancer cell proliferation, promotion of tumor angiogenesis, and extracellular matrix remodeling. Exosomes could also promote tumor drug resistance through a variety of mechanisms. Moreover, exosomes could help cancer cells evade immune surveillance and develop immune tolerance to evade attack by the immune system. The role of exosomes in tumorigenesis, metastasis, and drug resistance suggests that some new approaches could be developed for cancer diagnosis and treatment based on the nature of exosomes. Exosome-derived miRNAs can be obtained from a variety of bodily fluids, including urine, serum, plasma, saliva, and semen, making them valuable for large-scale cancer screening. Unlike tissue aspiration biopsy, which offers limited insight into a single tumor region at a specific time, exosome-derived miRNAs provide a solution to this limitation. On the basis of existing research, exosome-derived miRNAs not only complement current methods for grading and staging urinary system cancers but also hold promise in predicting tumor recurrence, metastasis, and evaluating treatment efficacy. This information aids patients in choosing the most suitable treatment approach (47). Exosome-derived cell-free DNA (cfDNA) and miRNA play crucial roles in tumor progression, drug resistance, and the regulation of the tumor microenvironment, establishing them as promising candidate biomarkers. Different from tissue aspiration biopsy, it is easy to obtain liquid biopsies and isolate exosomes from patients with cancer. In the future, more and more studies will focus on the role of exosomes in the early diagnosis of cancer. In clinical therapy, the ability of exosomes to regulate the tumor microenvironment allows them to be used as natural drug carriers with targeted properties that can be used to kill cancer cells without affecting healthy cells.

However, there remain notable limitations in current exosome research. Primarily, technical challenges persist in the separation and isolation of exosomes. Low-speed centrifugation, often employed for EV separation, lacks specificity and can introduce contamination from other membranous organelles. Conversely, high-speed centrifugation, while yielding higher quantities of exosomal miRNAs and proteins, produces different miRNA expression profiles compared to isolation kits with similarly high yields (48). This underscores the need for the development of more definitive exosome separation method. Furthermore, the mechanisms underlying exosome actions warrant further investigation. In tumor microenvironments, tumor cells release exosomes to modulate immune cell function, aiding in immune evasion. Tumor cells also utilize exosomes to transmit biological information among themselves, facilitating metastasis and drug resistance. Moreover, in highly vascularized cancers like renal cancer, the involvement of exosomes remains unclear. These findings highlight the promise of exosomes as potential targets for tumor therapy, suggesting that their biological properties could be further leveraged for drug development.

## Author contributions

RR: Writing – original draft. LZ: Writing – review & editing. SH: Writing – review & editing.
